# Effect of Welding Process Parameters on the Strength of Dissimilar Joints of S355 and Strenx 700 Steels Used in the Manufacture of Agricultural Machinery

**DOI:** 10.3390/ma16216963

**Published:** 2023-10-30

**Authors:** Jarosław Szusta, Łukasz Derpeński, Özler Karakaş, Nail Tüzün, Sławomir Dobrzański

**Affiliations:** 1 Faculty of Mechanical Engineering, Department of Machine Design and Exploitation, Bialystok University of Technology, Wiejska 45C Str., 15-351 Bialystok, Poland; j.szusta@pb.edu.pl (J.S.); l.derpenski@pb.edu.pl (Ł.D.); 2 Mechanical Engineering Department, Engineering Faculty, Pamukkale University, Kinikli, 20160 Denizli, Türkiye; 3 Department of Mechanical Engineering, Engineering Faculty, Istanbul Arel University, Tepekent, Buyukcekmece, 34537 Istanbul, Türkiye; nailtuzun@arel.edu.tr; 4 SaMASZ Sp. z o. o., Trawiasta 1 Str., 16-060 Zabludow, Poland; slawomir.dobrzanski@samasz.pl

**Keywords:** dissimilar welds, high strength steel, structural steel, weld parameters, structural design

## Abstract

The paper evaluates the possibility of using dissimilar materials joined by welding technology in the construction of agricultural machinery. The desire to design larger and more efficient structures requires designers to combine materials with different mechanical and structural properties. In such a case, it is very important to properly select welding parameters so that, on the one hand, the quality of the joint meets the standard requirements, and on the other, the welding process is not too energy-intensive. In this paper, overlay joints connecting S355 steel with Strenx 700 steel were analyzed in terms of strength for three different values of welding parameters and different thicknesses. The starting point was the reference parameters recommended by the company’s welding technologists, which were reduced by 10 and 20% according to the linear welding energy. The study compared the strength, ductility and macrostructure of the joints, as well as the energy intensity of the process. The proposed dissimilar joints achieved approximately a 10% increase in the strength limit of the components in comparison to the previously recommended welding parameters. Additionally, finite element analysis calculations of the improved designs showed significant weight reduction (up to 40%) for the relevant agricultural machinery components.

## 1. Introduction

The development of agriculture is dictated primarily by the need to produce higher quantities of food while enhancing its quality. These changes are significantly influenced by new technologies and process innovations used in the production of machinery and equipment for agriculture. They contribute to an increase in the efficiency and productivity of agrotechnical operations. In the era of the widely understood Agriculture 4.0 linked to the balanced agriculture system, there is a trend toward the use of high-performance machinery with large working widths. Due to the steadily decreasing natural resources and growing global population, it is necessary to guide agricultural production toward more balanced and efficient use of natural resources. In order to ensure food security, the share of large and very large farms in the agro market is increasing. For their operation, these enterprises need high-performance, cost-effective, large-width machinery that allows precision farming. To meet the expectations of the agro market, manufacturers of agricultural machinery must use unconventional combinations of construction materials at the stage of designing high-performance machinery dedicated to specific agrotechnical operations, to ensure a high level of reliability and operational durability of machinery implementing the concept of Agriculture 4.0.

### 1.1. Agricultural Applications

In an era of rising agricultural costs and falling prices for agricultural products, the world is facing a new challenge. In particular, this challenge is the search for cost savings in the means of production of agricultural machinery, so as to provide the farmer with economical and relatively inexpensive solutions. In the case of cattle, horse, sheep and biomass producers for energy needs, to achieve increased efficiency and economy in the green fodder harvesting chain, it is necessary to provide machinery solutions with increased operating productivity while reducing the times of individual agrotechnical procedures. Cheaper, more economical and more efficient machines provide a lower depreciation threshold which allows for a faster increase in profitability and earned income. The time saved and increased income enable farmers to reallocate capital to other sectors of the farm.

**Figure 1:**
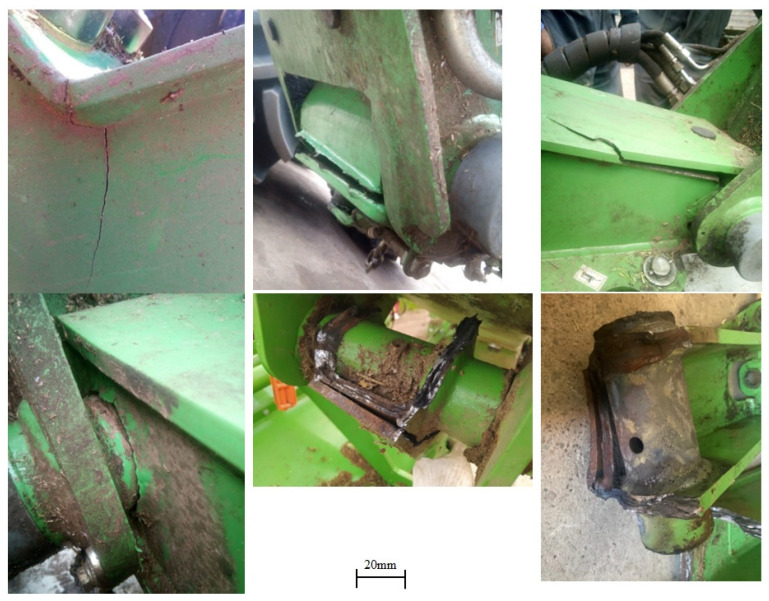
Operational cracks in forage harvesting machine structures made of S355 steel.

Rising production costs associated with, among other things, high prices of raw materials including steel, electricity and labor, force machine manufacturers to optimize manufacturing processes in order to provide the end user with a highly efficient, reliable product at an attractive price. Structures of high-performance machines made of typical construction materials, such as S355 steel, are subject to damage during extreme operation by cracking of their components. Examples of such damage can be found in the structures of green forage harvesting machines (Figure 1).

**Figure 2:**
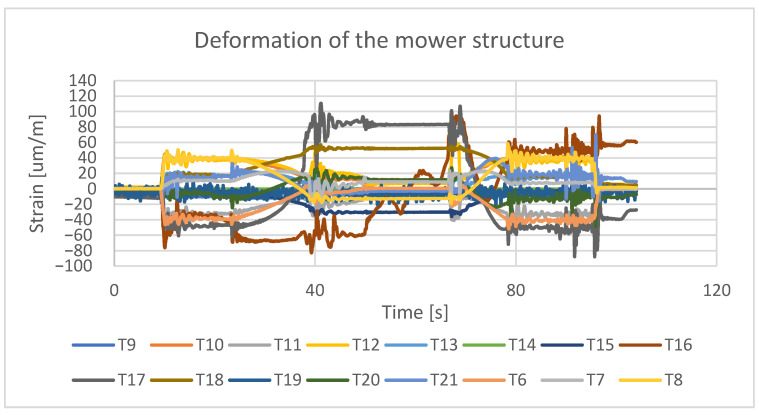
Example of the path of deformation in the structure of the mower during the operation of a green forage harvesting machine.

**Figure 3:**
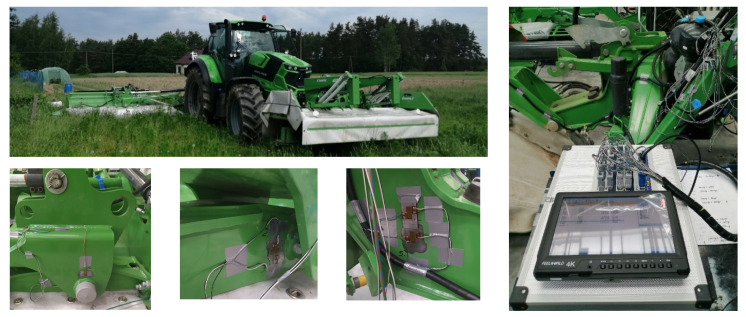
Test stand for determining operational loads during the grass-cutting process.

Typically, these cracks are fatigue cracks, caused by cyclic operating loads resulting from the variety of field layouts and the nature of agricultural tractor driving. Examples of the levels of operational loads occurring during the operation of the mower set are shown in Figure 2. The loads in question were acquired through strain gauge tests, involving the placement of 16 electro-resistive strain gauges in stress concentration regions of the machinery structure. This methodology allowed the real-time collection of deformation data during agrotechnical operations conducted in the field, as illustrated in Figure 3.

Welded joints in the context of high-strength steel materials have received significant attention from both scientific and industrial sectors, with applications extending to various fields, including agricultural machinery. The utilization of dissimilar welds, where different materials are carefully selected and joined, offers distinct advantages over constructing machinery from a single material. Incorporating dissimilar joints enables the use of materials with higher strength properties in localized stress concentrators without uniformly increasing section thickness, promoting a more even stress distribution and thus enhancing operational durability. This approach can yield machinery designs that achieve a delicate equilibrium between longevity, weight optimization, and cost efficiency. By carefully choosing materials for specific components or sections, engineers can utilize the superior properties of each material, such as strength, durability, or corrosion resistance, to meet the specific requirements of different parts of the machinery. This not only enhances the overall performance and longevity of the equipment but also allows for more efficient utilization of resources, making it a compelling choice for modern manufacturing processes. These advantages have contributed to the acceptance of dissimilar joints in industries such as shipbuilding, chemical, energy, automotive, and machinery, underscoring its practical and economic benefits.

Optimizing parameters is vital for any modern manufacturing method. However, to do so assumes even greater significance in welding processes, as these processes directly influence the mechanical behavior of materials due to variations in welding parameters. This is even more true for dissimilar joints, where the correct parameter adjustments are imperative to ensure the structural integrity and quality of the welds. These direct improvements to welding parameters enhance equipment performance and longevity, reduce maintenance costs and downtime, and conserve resources. Moreover, optimized parameters ensure high-quality, defect-free welds, ensuring structural integrity and product safety. In summary, parameter optimization is vital for efficient, cost-effective, and eco-friendly manufacturing processes.

### 1.2. State of the Art

Due to the previously elaborated considerations, the subject of dissimilar welds of high-strength materials has gained increasing importance in recent years. By extension, there has been a growing interest in the Strenx, a highly durable and lightweight steel, and its welded joints. In order to provide an overview of the current state of the research in this area, a short literature review was conducted, including studies on the welding of high-strength steels with a particular focus on Strenx 700 and their dissimilar welds.

Autogenous laser welding of Strenx 1100MC and laser-welded joints of Strenx 700MC steel [1,2] highlighted the influence of laser welding parameters, revealing the absence of cracking but a decrease in heat-affected zone (HAZ) microhardness, affecting tensile strength and ductility. In addition, research on thermomechanically processed high-strength steel Strenx 700MC [3] has examined its fracture behavior under impact loads and the influence of welding, revealing a specific fracture behavior influenced by internal structural heterogeneity. Laser butt joints made of DOCOL 1200 M martensitic steel sheets underwent comprehensive assessments of hardness, quasi-static and dynamic tensile tests [4]. The study provided essential comparisons with Strenx S700MC steel tests, offering insights into the mechanical performance of laser-welded joints. In [5], plasma + Metal Active Gas (MAG) welding of S700MC high-strength steel was examined with a focus on microstructure, hardness, and impact toughness, which led to this hybrid welding method being proposed as a viable option. Additionally, a thorough examination of residual stress and microstructural analysis in welded Strenx 700MC was performed in [6], revealing significant grain coarsening and tensile residual stress fields. A critical evaluation of Strenx 960MC welded joints [7] scrutinized the influence of welding parameters and filler materials on the heat-affected zone. Variations in heat input and cooling rates led to changes in the properties of the heat-affected zone, illustrating the balance required to optimize mechanical properties. Hybrid laser-arc welding for high-strength steels was examined in [8], demonstrating successful weld samples prepared from S690QL steel. The study highlighted the process’s potential for high-strength steels but mentioned non-negligible interpass temperatures for the second weld. Charpy impact tests on Strenx 700MC steel and its weld were detailed in [9]. The study observed the influence of impact energy on material properties and fracture zones. Furthermore, the study addressed the critical issue of the welding heat input’s influence on residual stress and welding deformation in large, thin-walled constructions made of S690 high-strength steel [10]. Optimizing welding parameters, especially welding speed, was shown to significantly reduce deformation and improve production precision. In [11], the authors aim to investigate the mechanical properties of welded joints made of carbon steel A48 AP and explore the factors contributing to fatigue and structural failure. The study employs a global (J and CTOD) and a local (Rice-Tracey model) fracture mechanics approaches to understand the impact of defects and stress changes on the welded joint’s integrity and susceptibility to damage. The same material is further examined in [12] where the focus is on the numerical simulation of axisymmetric notched specimens to examine nucleation using the Gurson–Tvergaard–Needleman model (GTN). The simulations involve the application of the GTN model to describe material damage, taking into account stress triaxiality. On a different note, ref. [13] emphasizes the importance of effectively controlling materials development processes and adapting efficient assembly methods. Specifically, it discusses the challenges and intricacies of friction stir welding for joining aluminum alloys like AA6061-T651 and AA7075-T651. The study highlights the need to optimize mechanical properties, especially high rupture strength in tensile tests, by controlling various parameters such as welding speed, rotation speed, and pin type.

Evaluations that were extended to dissimilar welding by performing quality assessments of dissimilar welded joints of Hardox 450 and Strenx 700 involving non-destructive testing and microstructural analysis unveiled the significance of welding consumables in achieving the required quality of welded joints [14]. Moreover, the weldability of ultra-high-strength steel and advanced high-strength steel was examined [15] by investigating the welds of S700MC/S960QC, which further demonstrated the importance of precise welding processes and parameters. The impact of welding heat input parameters on the microstructure and hardness of the heat-affected zone in the dissimilar welds of quenched, tempered and thermomechanically controlled process 690-MPa high-strength steels was investigated in [16]. Through numerical analysis and experiments, the paper examined temperature variations and their impact on heat-affected zones in dissimilar welded joints and provided valuable insights into optimizing welding parameters for improved HAZ characteristics. The review article [17] summarizes the microstructure and mechanical behavior of dissimilar welded joints between ferritic–martensitic steel and austenitic stainless steel in power plants. The filler wire selection, post-weld heat treatment and the effects of residual stress on component failure are explored, highlighting methods to improve the mechanical properties of dissimilar welded joints. Moreover, the experimental study [18] investigates the impact of weld groove geometry, narrow V groove and double V groove, on Alloy 617/P92 steel dissimilar metal weld joints. Microstructure and hardness varied along the weld, with the peak hardness in the coarse-grain heat-affected zone of P92 steel. Tensile tests confirmed joint applicability for ultra-supercritical applications but with lower strength compared to base metals. In [19], a comprehensive literature review is presented, highlighting recent advancements in understanding mechanical properties, such as fatigue strength, tensile strength, and hardness in dissimilar welded joints. The study explored various welding techniques for joints involving metals with different types and compositions, focusing on evaluating their fatigue behavior and the impact of chemical composition on weldability and mechanical properties.

The impact of varying heat input on the microstructure and mechanical properties of dissimilar high-strength steels (S700MC/S960QC) joined using undermatched filler material and gas metal arc welding was investigated in [20]. The research experimented using different heat input values and revealed that a specific heat input resulted in improved microstructure formation and mechanical properties in the heat-affected zone of both materials. Furthermore, the study in [21] explored the welding of S960QL steel and S304 steel, emphasizing the advantages of laser welding techniques in reducing heat-affected zones and hot cracking tendencies. Notably, the research produced good-quality joints with minimal imperfections and favorable mechanical properties. The research in [22] evaluated the dissimilar joints of S690QL high-strength steel pins and arms made of DOCOL 1200M steel. The study investigated this challenging welding process, proposing welding parameters and filler materials that ensure proper joint formation without welding defects. Investigating strength mismatch modes in dissimilar welded joints of Q390 and Q690 steels, [23] highlighted the significance of microstructural characterization and mechanical testing. The study proposed an acceptable strength mismatch for dissimilar welded joints with acceptable mechanical properties. In another paper [24], the research focused on the microstructure evolution within dissimilar weld metal, specifically the local brittle zone, in welded joints of S690QL high-strength steel. Multiple thermal cycles and base metal element dilution were found to influence the microstructure and impact toughness of the root zone.

In summary, this literature review provides a brief examination of the current state-of-the-art in welding high-strength steel materials, covering various welding processes, parameters, and their impacts. However, it is evident that there exists a noticeable gap in the literature regarding the investigation of dissimilar welds involving Strenx 700. Recognizing this gap, the present research paper aims to contribute significantly to this area by addressing the limited knowledge available on the welding of Strenx 700 with dissimilar materials, specifically focusing on optimizing the welding parameters necessary for joints with desirable mechanical properties.

### 1.3. Novelty

In order to ensure an adequate level of reliability in the design of agricultural machinery for harvesting green fodder, research is needed to reduce the weight of machines while increasing their strength and stiffness. Therefore, there is a reasonable need to look for solutions that increase the load-to-weight ratio and reduce production costs.

To achieve this goal, the study initially focuses on attaining mechanical reliability in dissimilar joints. Once joints with adequate mechanical properties are achieved using the recommended welding parameters, the study investigates the mechanical properties of joints that were welded with lower welding energy. This approach effectively reduces production costs by minimizing energy consumption while still enhancing the mechanical properties of the welded joints.

The improved design of currently available agricultural machinery is realized through the implementation of these dissimilar joints and welding parameters, resulting in a significant reduction in weight without sacrificing structural reliability.

## 2. Materials and Methods

**Figure 4:**
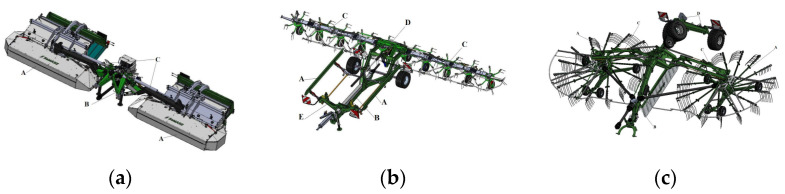
Technological sequence for harvesting green fodder from multi-plots using Samasz machinery: (**a**) mower set; (**b**) tedder; (**c**) rake.

In order to reduce the energy consumption of the welding process of machine components manufactured by the company Samasz, Zabludow, Poland, the first step was to analyze the types of welded joints used during the construction of typical machines: a disc mower (Figure 4a), a rotary tedder (Figure 4b) and a rake (Figure 4c).

The solutions for the structural nodes of the analyzed machines, which are particularly prone to deformation, are designed as framed-box structures. These structures are crafted using welding techniques with formed steel sheets. The most common choice for the structural material in these solutions is S355 steel, which is frequently susceptible to damage resulting from local overloads (Figure 1). Increasing the thickness of the sheet is a short-term solution that does not provide sufficient operational reliability in the long term. Therefore, it was decided in the present research to use dissimilar materials in the structural nodes of machines, the combination of which would provide the required strength and stiffness along with weight reduction. The critical point in such a combination of materials is the technological joining process, which can ensure optimal properties of the connection. For the construction of the machine elements that are under the highest loads, a combination of S355 steel and Strenx 700 (SSAB AB, Stockholm, Sweden) was proposed. Unsurprisingly, the fusion of materials with notably distinct grades necessitates a new approach to their joining process, primarily stemming from the twofold difference in yield strength between the two materials. The combination of low-cost structural steel of ordinary quality S355 with the high-strength steel Strenx 700 ensures the production of machinery with higher durability, weight reduction, fuel efficiency and extended service life. In addition to the overall improvement in design, another crucial point to consider is the long-term agricultural benefits that stem from this innovative material combination. The increasing average farm size with the growing demand for intensive agro-technical work is forcing the production of larger and heavier machinery. These machines can cause soil compaction and long-term yield reduction. Therefore, sustainable agriculture should use high-strength steels to offer lighter, stronger and more efficient solutions. Specialty steels in the form of Strenx can help reduce the weight of agricultural machinery by up to 30–50% without sacrificing durability. When combining the two materials, it is difficult to determine the optimal welding parameters without appropriate experimental examinations. Therefore, this paper attempts to determine the optimal industrial joining parameters of the analyzed material grades in terms of ad hoc strength and welding economics.

Based on an analysis of machine design, Table 1 summarizes a proposal for the types of welded joints, thicknesses and types of sheet metal planned for use on structural nodes of green forage harvesting machines.

Table 2 shows the strength properties of the two materials being joined. Additionally, the chemical composition of the jointed materials is shown in Table 3. The analysis was conducted utilizing a Hitachi S-3000N (Hitachi Ltd., Tokyo, Japan) scanning electron microscope equipped with an X-ray microanalysis device of the Quest type from Thermo Noran (Thermo Fisher Scientific Inc., Waltham, MA, USA).

### 2.1. Preparation of Test Specimens

In order to carry out the planned experiments, the test joints from dissimilar materials (S355 steel, Strenx 700) were prepared. The base material was structural steel S355 in the thickness range of 6–14 mm, sheets of which were joined by double-sided overlays made of a 5 mm thick sheet of Strenx 700 material. Welds were made using MAG technology. A fusible electrode in the form of Lincoln Supramig HD ISO 14341-A-G46 4 M21 wire was used for welding [25]. The welding process was performed with the parameters listed in Table 4. For the test joints, the recommended parameters of the welding technicians were used as the V1 variant. As for the V2 and V3 variants, less than the recommended parameters were tested, so that the linear welding energy was 90%V1 and 80%V1, respectively.

**Figure 5:**
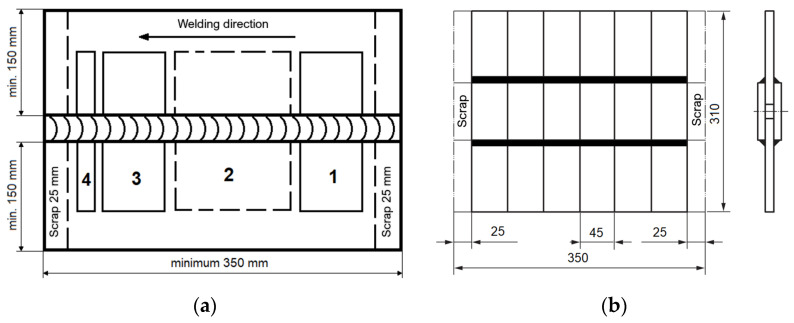
Arrangement of test specimens on the test joint: (**a**) butt weld: specimens for tensile (1, 3), bending (1, 3), impact test (2) and hardness measurement (4); (**b**) overlay weld.

Figure 5 shows the arrangement of specimens for destructive testing on a welded test joint according to EN ISO 15614 and EN ISO 15609 [26,27].

**Table 4:** MAG welding parameters of welded joints.

Sample 1 [S355 #5 + Strenx #5]				
Parameter	Value	V1	V2	V3
Current amperage	A	215	175	160
Current voltage	V	21	19.5	16.5
Type of current		DC+	DC+	DC+
Welding speed	m/min	0.26	0.22	0.19
Wire feed speed	m/min	12	10	9
Welding material		Lincoln Supramig HD ISO 14341-A-G46 4 M21		
Binder diameter	mm	1		
Shielding gas		M23 (90% Ar; 5% C02; 5% 02) ISO 14175 [28]		
Gas flow rate	L/min	14–16		
Amount of heat input	kJ/mm	0.83	0.74	
S355 #5 Strenx #5	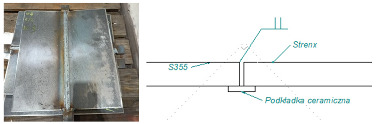			
**Sample 2 [S355 #6 + Strenx #5]**				
**Parameter**	**Value**	**V1**	**V2**	**V3**
Current amperage	A	250	200	180
Current voltage	V	25	23	19
Type of current		DC+	DC+	DC+
Welding speed	m/min	0.38	0.31	0.26
Wire feed speed	m/min	15	13	11
Welding material		Lincoln Supramig HD ISO 14341-A-G46 4 M21		
Binder diameter	mm	1		
Shielding gas		M23 (90% Ar; 5% C02; 5% 02) ISO 14175		
Gas flow rate	L/min	14–16		
Amount of heat input	kJ/mm	0.64	0.58	0.55
S355 #6 Strenx #5	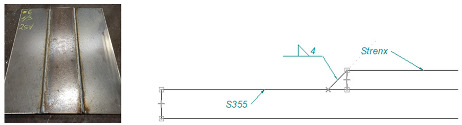			
**Sample 3 [S355 #10 + Strenx #5]**				
**Parameter**	**Value**	**V1**	**V2**	**V3**
Current amperage	A	255	225	185
Current voltage	V	26	23.5	20
Type of current		DC+	DC+	DC+
Welding speed	m/min	0.48	0.43	0.33
Wire feed speed	m/min	255	225	185
Welding material		Lincoln Supramig HD ISO 14341-A-G46 4 M21		
Binder diameter	mm	1		
Shielding gas		M23 (90% Ar; 5% C02; 5% 02) ISO 14175		
Gas flow rate	L/min	14–16		
Amount of heat input	kJ/mm	0.63	0.59	0.49
S355 #10 Strenx #5	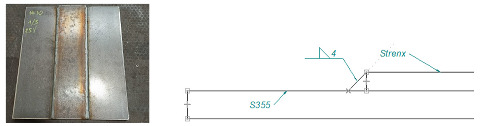			
**Sample 4 [S355 #12 + Strenx #5]**				
**Parameter**	**Value**	**V1**	**V2**	**V3**
Current amperage	A	275	240	195
Current voltage	V	27	24.5	21
Type of current		DC+	DC+	DC+
Welding speed	m/min	0.58	0.51	0.4
Wire feed speed	m/min	275	240	195
Welding material		Lincoln Supramig HD ISO 14341-A-G46 4 M21		
Binder diameter	mm	1		
Shielding gas		M23 (90% Ar; 5% C02; 5% 02) ISO 14175		
Gas flow rate	L/min	14–16		
Amount of heat input	kJ/mm	0.61	0.59	0.51
S355 #12 Strenx #5	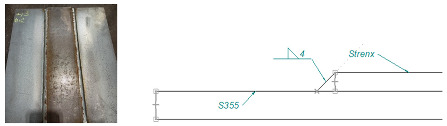			
**Sample 5 [S355 #14 + Strenx #5]**				
**Parameter**	**Value**	**V1**	**V2**	**V3**
Current amperage	A	280	250	220
Current voltage	V	28	25	21.5
Type of current		DC+	DC+	DC+
Welding speed	m/min	0.47	0.42	0.35
Wire feed speed	m/min	17	14	12.5
Welding material		Lincoln Supramig HD ISO 14341-A-G46 4 M21		
Binder diameter	mm	1		
Shielding gas		M23 (90% Ar; 5% C02; 5% 02) ISO 14175		
Gas flow rate	L/min	14–16		
Amount of heat input	kJ/mm	0.47	0.42	0.35
S355 #14 Strenx #5	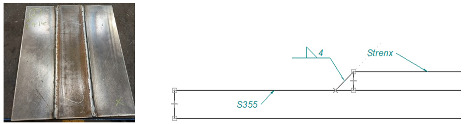			

**Figure 6:**
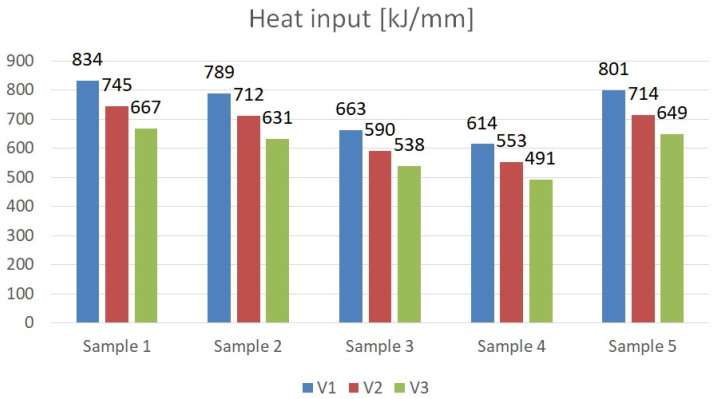
Comparison of welding line energies for the adopted parameters and material grades.

Strenx steel is a high-alloyed material with a fine-grained structure, unlike S355 steel. Using welding parameters dedicated to one of these two materials can adversely affect the properties of the joint. Therefore, through the experimentation of the linear welding energy parameter, the optimal values of current, voltage and welding speed were determined for the adopted quality criterion which is the strength of the joint. The welding linear energy (Figure 6) was determined for the manufactured joints and welding parameters according to Equation (1):

(1) $$Q=\frac{IU}{V1000}k$$

where: *I*—average value of welding current [A], *U*—average value of arc voltage [V], *V*—welding speed [mm/s], *k*—thermal efficiency factor (for the parameters used, *k* = 0.8).

**Figure 7:**
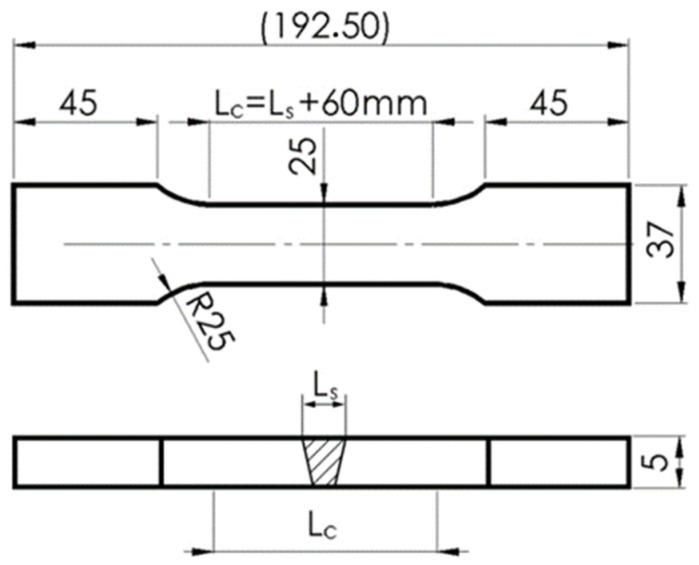
Sample dimensions for monotonic tension test, butt weld (all dimensions are in mm) (EN ISO 4136).

**Figure 8:**
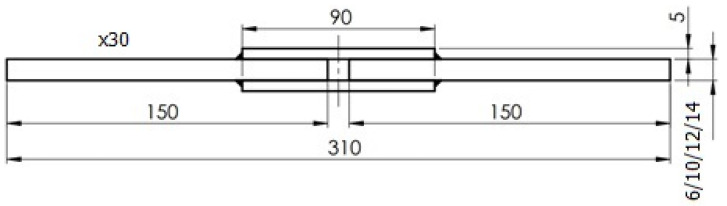
Sample dimensions for monotonic tension test, overlay weld (all dimensions are in mm) (PN-EN ISO 9018).

For all the welding parameters analyzed, the joints that were prepared have passed visual examinations. The welded joints underwent a visual inspection (non-destructive testing) following the application of specific welding parameters prior to the final sample cutting. This inspection encompassed an assessment of the weld’s appearance, geometry (dimensions), quality, shape, surface condition, and the presence of potential welding irregularities such as open cracks, assembly discrepancies, delaminations and adhesions, as well as any discernible bubbles and inclusions. In all analyzed instances, plates welded with the parameters designated in this study consistently met the established criteria, making them suitable for the extraction of the requisite test samples. Waterjet cutting technology was utilized to ensure correct shapes for the sample joints, and specimens that were used in the strength tests were prepared in accordance with Figure 7 and Figure 8. Two different static tensile test samples were prepared: butt joints (Sample 1) were prepared according to PN-EN ISO 4136:2022-12 [29], while overlay joints (Sample 2, 3, 4, and 5) were prepared in compliance with the PN-EN ISO 9018:2016-01 standard [30].

### 2.2. Test Stand

**Figure 9:**
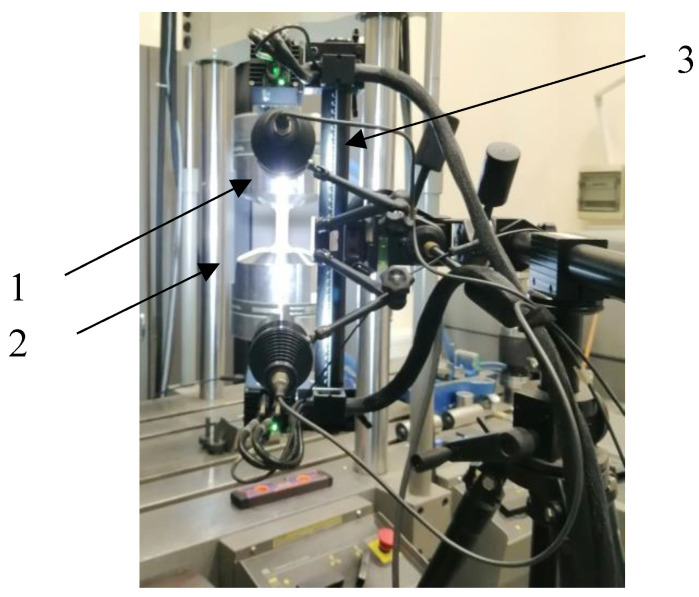
Sample on the test stand.

The experimental setup is shown in Figure 9. The specimens (1) were subjected to loads in monotonic tensile tests with a constant speed of Δ*l* = 0.001 mm/s. An MTS 828 servohydraulic testing machine (2) was used for the tests. Measurement of specimen strain was carried out using an Aramis 4M vision system (3). During the test, the force and displacement of the specimen’s measurement base were recorded continuously. In addition, using the ARAMIS 4M vision system, the deformation of the specimen during load build-up was observed using the digital image correlation (DIC) method. Using the Aramis 3D 4M made it possible to register the deformation process and to locate critical failure deformations.

The tests conducted were aimed at determining the strength characteristics of the welded joints obtained, as well as determining the strain distributions in the test specimens analyzed. Each test was repeated three times, and the result was the arithmetic average of these repetitions. Similar to the geometry of the samples, the testing procedures followed the guidelines specified by the respective standards of PN-EN ISO 4136:2022-12 for butt joints and PN-EN ISO 9018:2016-01 for overlay joints.

## 3. Results and Discussions

**Figure 10:**
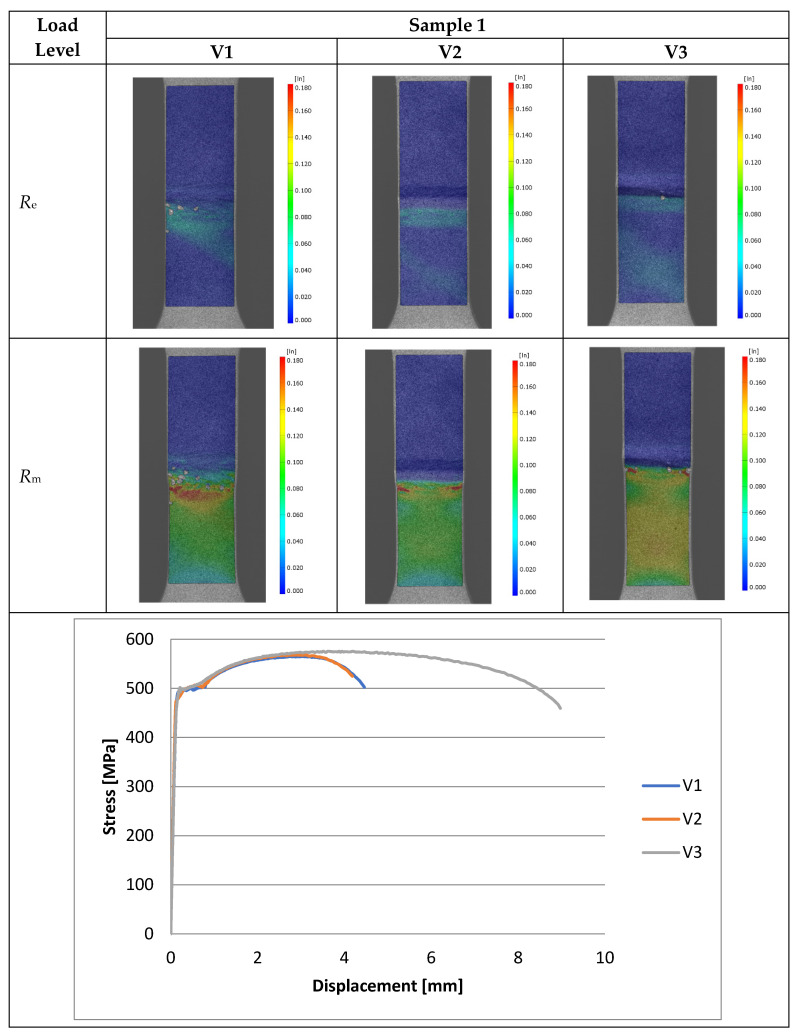
Tensile specimens with butt welds (Sample 1).

**Figure 11:**
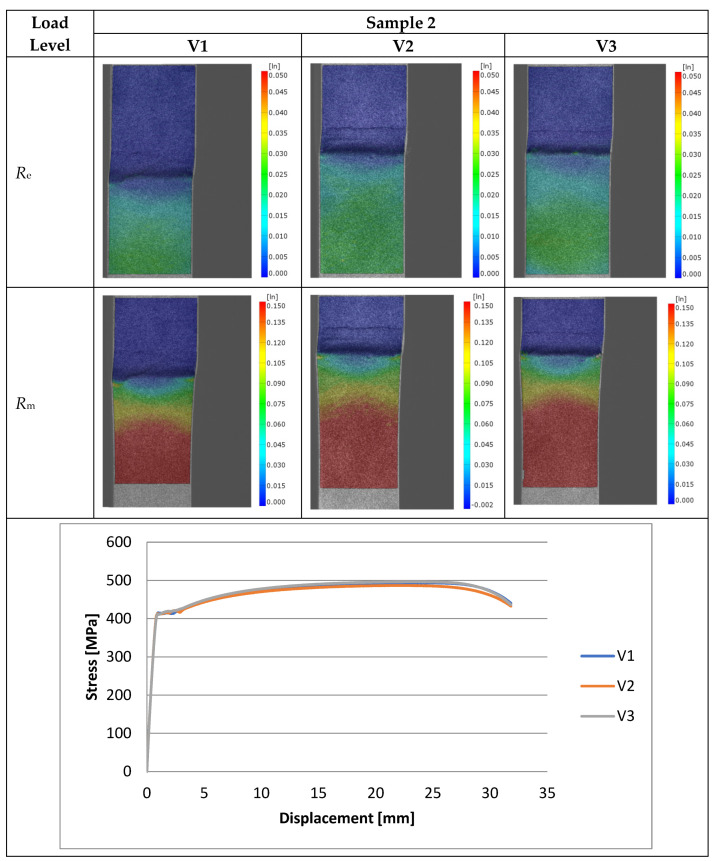
Tensile specimens with an overlay joint (Sample 2).

**Figure 12:**
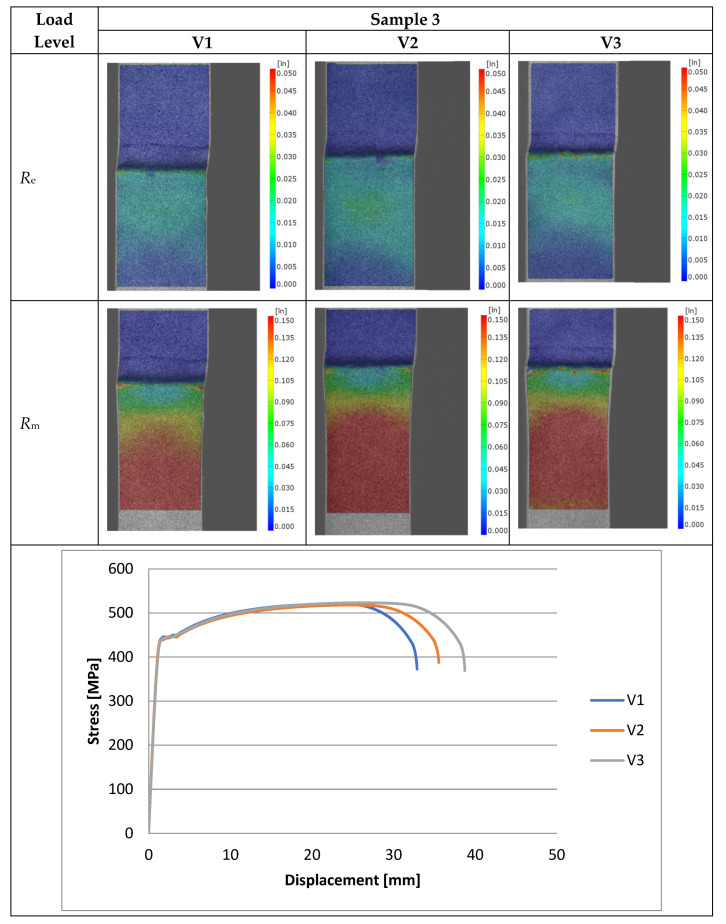
Tensile specimens with an overlay joint (Sample 3).

**Figure 13:**
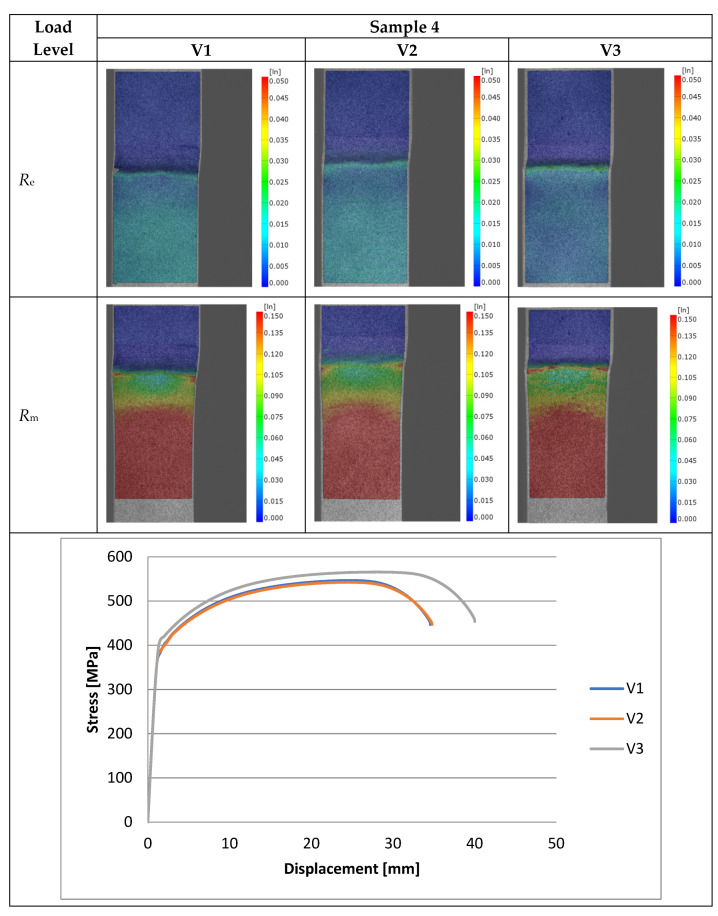
Tensile specimens with an overlay joint (Sample 4).

**Figure 14:**
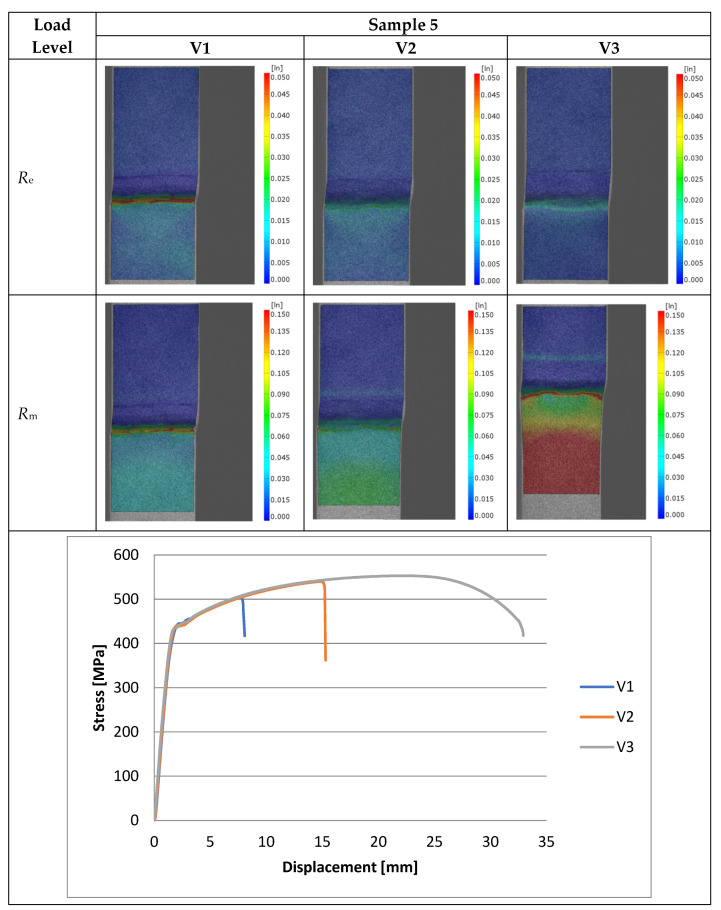
Tensile specimens with an overlay joint (Sample 5).

Experimental investigations consisted of determining the effects of the process and welding parameters on the mechanical properties of the material, including tensile strength (*R*_m_), yield strength (*R*_e_) and specimen elongation to break (*A*%). In addition, the fracture of the samples itself was analyzed to detect weld inconsistencies such as cracks, undermelting, fusion deficiencies, slag, and blister nests. Based on the observation of the fracture area, the appropriateness of the selection of the materials to be joined and the choice of welding parameters were determined. Figure 10, Figure 11, Figure 12, Figure 13 and Figure 14 show the results from the tests. Based on the stress versus displacement relationships obtained for the analyzed types of joints and welding parameters V1, V2, and V3, we can conclude that when the welding parameters are reduced from level V1 to level V3, there is no decrease in the temporary strength of the joint, and the material’s elongation at the point of failure increases, all while reducing the energy consumption of the welding process. Notably, the yield strength and Young’s modulus remain unchanged, while the joint exhibits increased ductility.

The test results conducted on the recorded deformations at the interface where two structurally distinct materials are joined reveal that, as heat input decreases, there is an increase in the material’s ductility, leading to a shift towards a more ductile form of fracture. This shift is a favorable phenomenon for the functioning of agricultural machinery.

**Figure 15:**
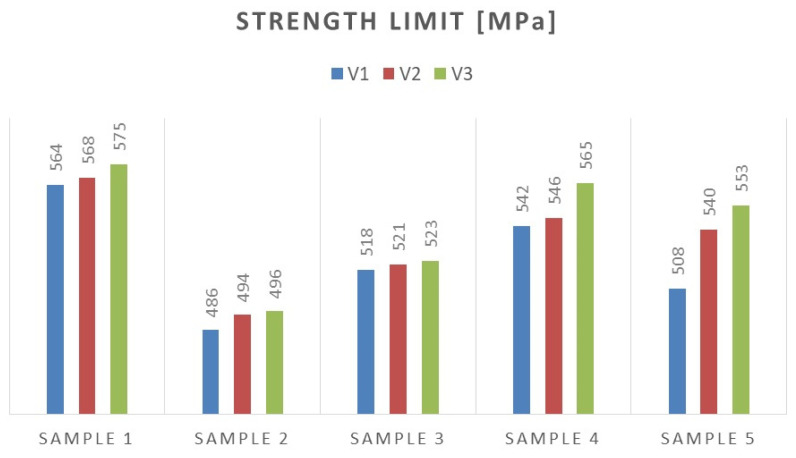
Comparison of tensile strength values of specimens made with given welding parameters.

**Figure 16:**
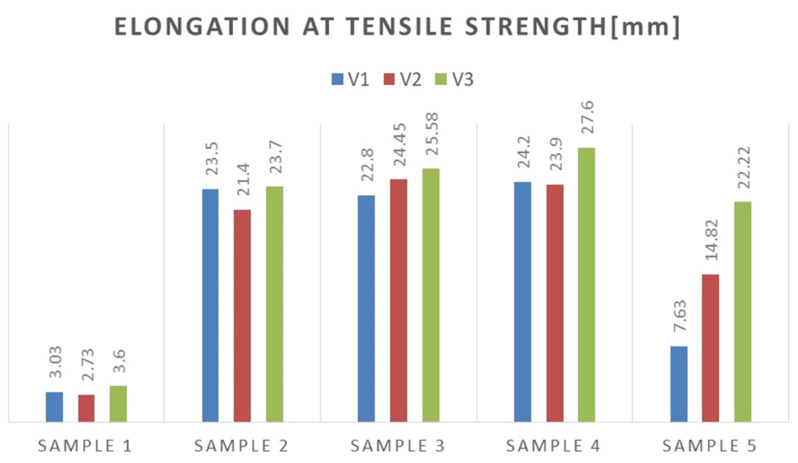
Compilation of elongation values of specimens corresponding to tensile strength limit.

Based on the performed tests, the tensile strength limit of the joint was found to be dependent on the welding parameters as demonstrated in Table 5. A comparison of mechanical properties between the welded joints and the base materials reveals that the ultimate tensile strengths of the welded joints are generally lower than Strenx but higher than S355. This outcome is consistent with the behavior expected from dissimilar welds, where joint strength aligns more closely with the material of lower strength. However, it is worth noting that most joints exhibit ultimate tensile strengths similar to or higher than S355, except for Sample 2. These variations can be attributed to changes in material properties and differences in intermetallic areas due to weld heat input. The highest strength was characterized by joints in which the welding parameters were reduced by 20% compared to the recommended values as illustrated in Figure 15. Moreover, reducing the welding parameters improved the ductility of the manufactured joint, as shown in Figure 16.

**Figure 17:**
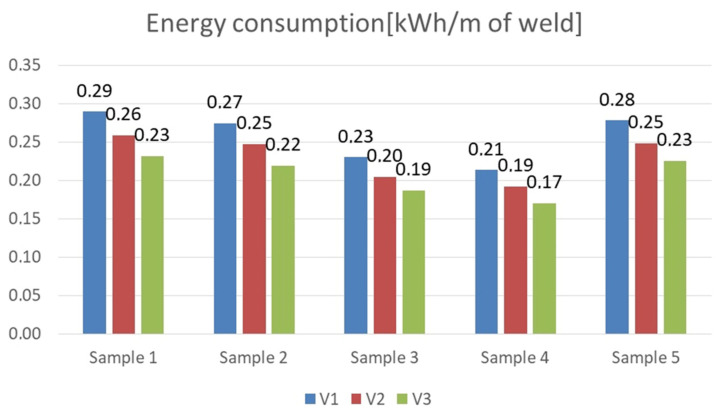
Energy consumption when welding 1 mm of weld depending on the adopted welding parameters.

The undoubted advantage of decreasing the welding parameters to a level that ensures the production of a high quality weld and satisfactory strength is the reduction of the energy intensity of the process (Figure 17), which brings measurable benefits to the production plant in terms of energy consumption, in addition to its good mechanical properties.

### 3.1. Microhardness Tests of the Joint

The distribution of microhardness in a dissimilar metal welded joint made of materials with differing strength and structural properties has a major impact on the performance of the obtained joint. In the analyzed example, S355 steel with a nominal hardness of 152 HV is joined with Strenx 700 steel with a hardness of 270 HV. As a result of the welding process, the bonded materials undergo induced structural changes related to the influence of high temperature and the diffusion of the binder, which becomes evident in the distribution of the microhardness of the joints. This study analyzed the effect of welding parameters on the microhardness distributions of an overlay and butt joint. The microhardness of the fabricated joints was measured using a Vickers microhardness meter (Sinowon, Guangdong, China). The results of the study are shown in Table 6 and Table 7.

Based on the ground microhardness profiles of the weld joint, intermetal areas with increased hardness near the fusion zone on the S355 steel side were observed. This transition from ductile to brittle fracture behavior is a critical concern, especially in the context of high load variations in agricultural machinery, posing a significant safety risk. In the boundary zone between the native Strenx material and its heat-affected zone, there is a local 10% reduction in microhardness compared to the original material. This reduction enhances the material’s ductility in the zone of large structural changes, which is a desirable phenomenon. Similarly, reducing the welding parameters by 20% (V3) in terms of linear welding energy compared to the recommended welding parameters of the V1 joint results in a decrease in microhardness in the heat-affected zone by 2%.

### 3.2. Microstructural Studies

**Figure 18:**
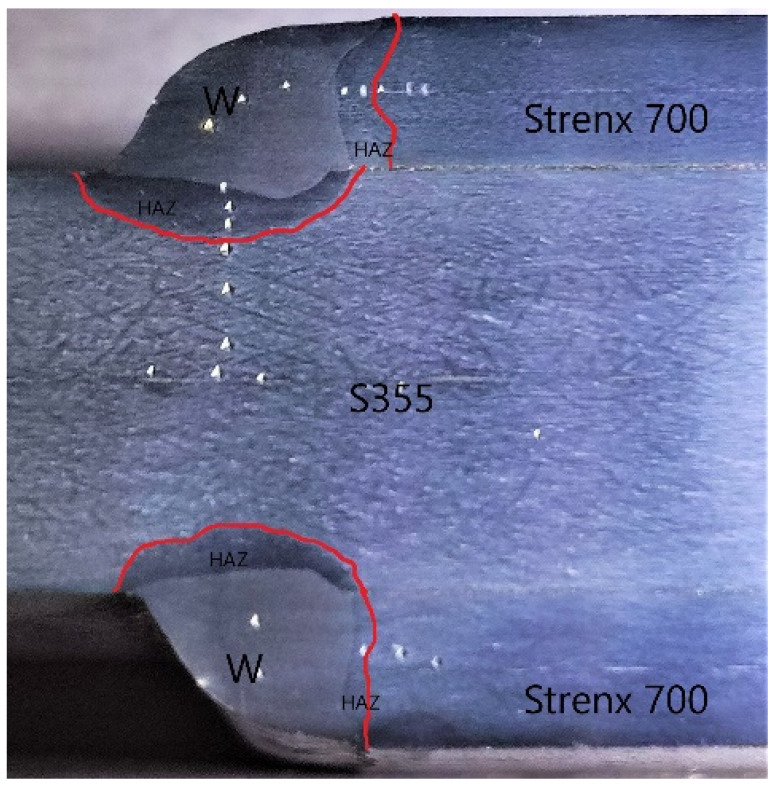
View of the joint with the welded joint components listed.

Figure 18 shows the heat-affected zones in both materials, marked with red lines. As can be observed, a larger area of the heat-affected zone is observed in the S355 material. Strenx 700 steel absorbed considerably less heat, so the structure of the material did not undergo a thorough remodeling.

By analyzing the structure of the obtained welded joint and relating the size of the HAZ to the size of the areas of the joint obtained with the reference parameters, it can be concluded that reducing the welding parameters by 10% results in a reduction in the size of the HAZ in the analyzed joint on the S355 steel side by 5%, and on the Strenx 700 side by 10%. Reducing the welding parameters by 20% results in a reduction of the heat-affected zone by 25% on the S355 side and by 20% on the Strenx 700 side. Table 8 compares the size of the HAZ for the analyzed welding parameters.

**Figure 19:**
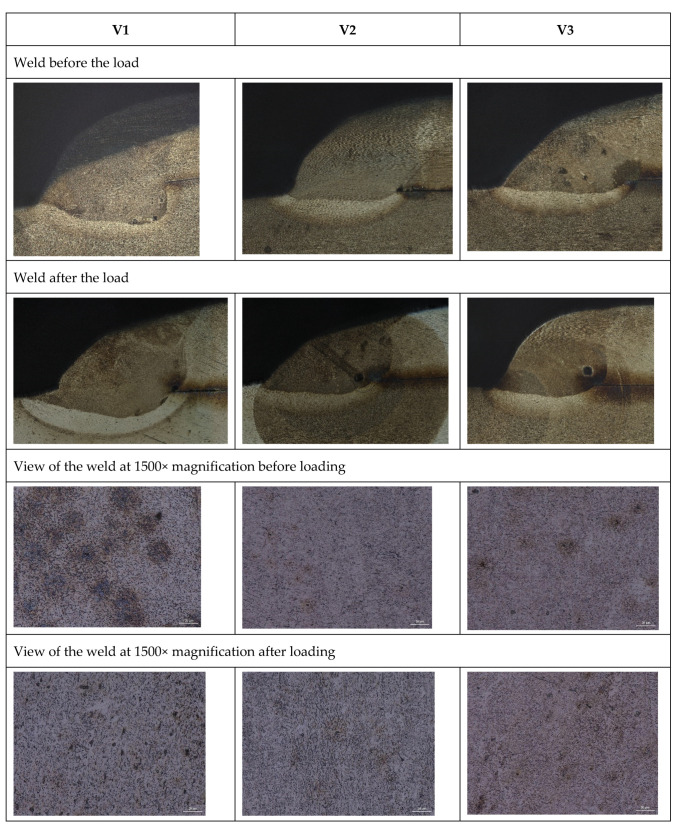
Macrostructure and microstructure of an overlay welded joint of dissimilar materials for different welding parameters (Sample 4).

Figure 19 displays the macrostructure and microstructure of the joint created using various welding parameters, along with the visual characteristics of the weld before and after tensile deformation. For all analyzed welding parameters, the weld keeps its integrity in the loading sequence, which indicates a well-made joint. Only slight grain deformation along the length due to the applied load is observed. As the welding parameters are decreased, the weld structure develops a finer grain size, resulting in the ability to carry a higher load.

**Figure 20:**
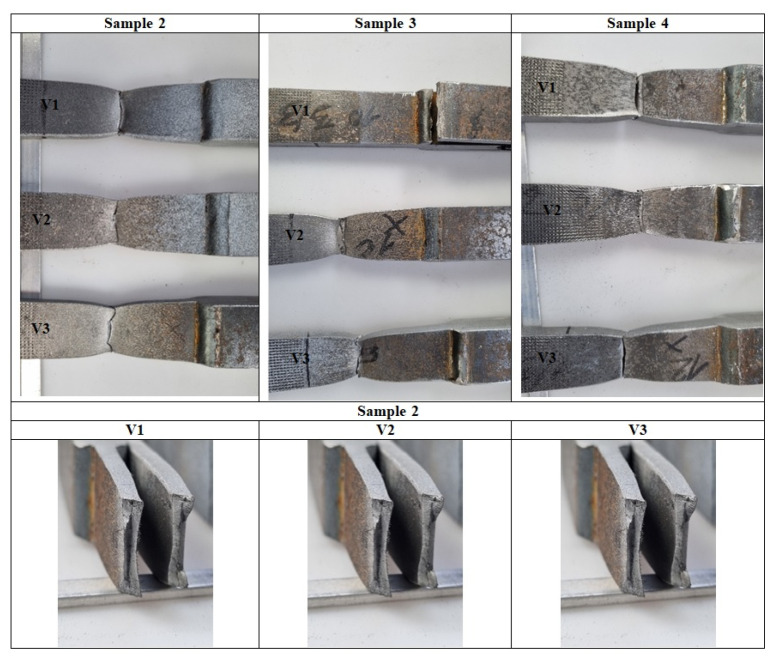
Views of specimen fractures obtained during tensile testing (Sample 2).

Figure 20 shows selected fractures obtained during static tensile testing. In all cases, fracture occurred in the S355 material outside the heat-affected zone. As expected for this material, the specimen deposits had a multi-planar structure.

### 3.3. Material Optimization of Agricultural Machinery Construction

**Figure 21:**
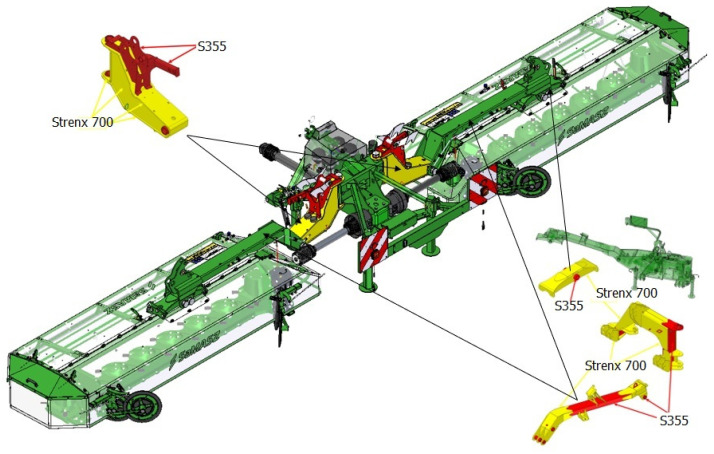
Selected structural nodes of green forage harvesting machines using dissimilar joining materials with selected welding parameters.

Subsequently, based on optimized welding parameters, structural nodes were selected in machines in which, in order to increase their ad hoc strength and reduce weight, joints of dissimilar materials were used with selected welding parameters representing 80% of the value of the recommended welding linear energy. Figure 21 shows selected redesigned nodes in machines using dissimilar materials (S355 and Strenx 700).

Redesigned according to these recommendations, the machines are currently in the field testing phase and, for the time being, show increased operational reliability.

Meanwhile, to confirm the assumptions made about the use of dissimilar materials in the construction of agricultural machinery, numerical calculations using finite element analysis (FEA) were carried out for the selected mower made by Samasz. A spatial discrete FEA model of mower constructions was made, taking into account all components affecting their stiffness and strength. Due to the specifications of the structural elements, the discretization was carried out using plate-shell elements (Quad4, Tria3) characterized by linear displacement functions and linear beam elements (CBEAM2). Separable connections were modeled using CBEAM2 elements and multi-point constrain (MPC) elements of RBE2 and RBE3 types. Calculations were performed using MSC software AFEA (Marc v2020). The preliminary results of the numerical calculations have been presented to demonstrate the uniformity of stress levels within the structure when different material grades are selected and applied. It has been observed that, subsequent to the modifications, stress values throughout the structure generally exhibit consistency, albeit with localized concentrations attributed to the structural design. The process of selecting different material grades also necessitated adjustments to the thickness of individual structural components, inadvertently resulting in a reduction in the overall stiffness of the calculated model. Consequently, modifications were implemented within the spatial structure of the model, including adjustments to the dimensions of the closed profiles created for the subject structure.

It is imperative to emphasize that the FEA calculations presented in this study are of a preliminary nature, primarily intended to illustrate the stress behavior of components manufactured from dissimilar materials. For a more comprehensive FEA, a more extensive approach to finite element modeling would be required, considering the regions with elements composed of varying material grades within the structures under examination and the changes in the stiffness of the calculated construction. However, this extends beyond the scope of the current paper and remains a subject for further research consideration by the authors.

**Figure 22:**
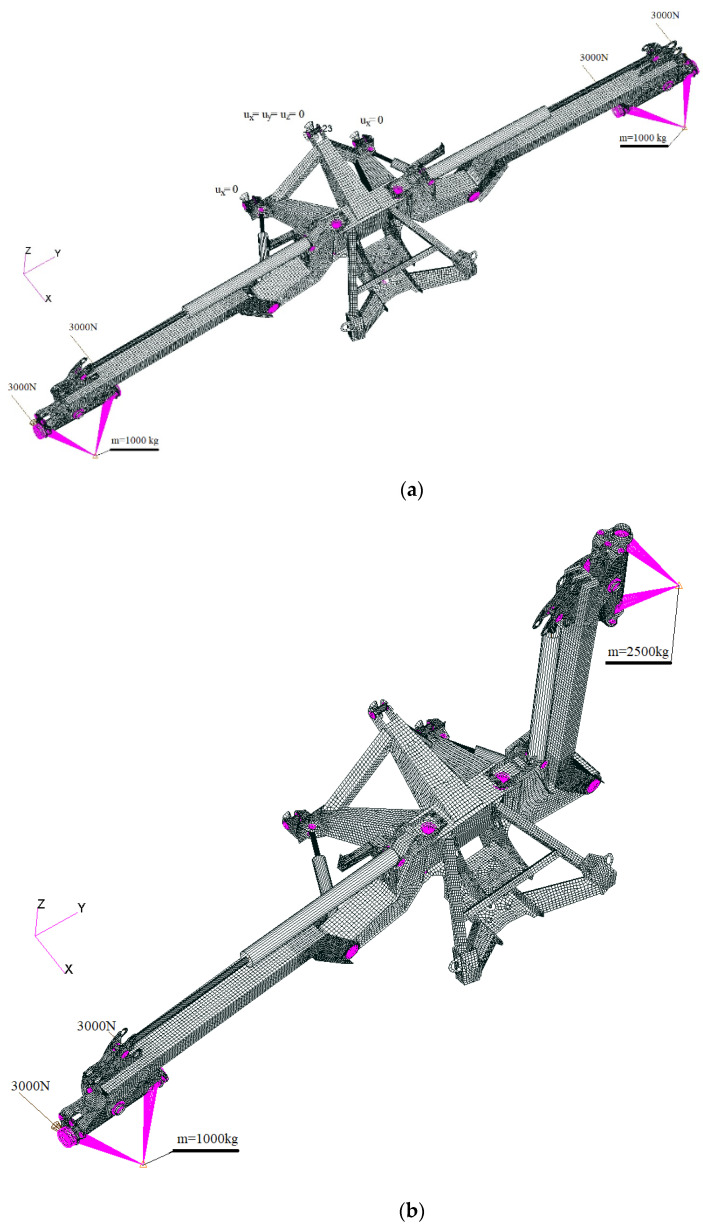
Discrete models of the main load-bearing components: (**a**) configuration 1; (**b**) configuration 2.

Figure 22 shows the discretized model in two loading configurations. Configuration (a) shows the mowing process, where a tractor-mounted mower is pulled across a field. Configuration (b) presents an asymmetrical unfolding of the mowing unit.

### 3.4. Boundary Conditions and Load Cases

Boundary conditions for individual models of discrete structures were adopted in accordance with their working conditions (Figure 22). In all models, translational boundary conditions were adopted. In the case of the mower machine, two configurations of the computational model (arms spread out, arms folded asymmetrically) were adopted. The load from the mower unit was taken into account in the form of a concentrated mass of 2500 kg applied to the center of gravity of the unit. In the first configuration, both mowing units are located on the ground, carry out the mowing process, and are moved along the ground. As a result, it was assumed that the impact of the mower units on the frame is reduced to 40% of the value derived from the unit weight (reduced mass m = 1000 kg). In addition, as a result of movement, the mower frame is subjected to a load from the friction of the mower unit sliders on the ground and is directed opposite to the movement. This impact was applied evenly in the form of a concentrated force of 6000 N to the node holding the mower unit. The second configuration of the computational model includes setting one of the arms in a vertical position. In this configuration, it was assumed that a concentrated mass of 2500 kg acts on the raised arms. Additionally, the arm placed in a horizontal position is loaded with the mowing unit impact weight reduced to 40% of the value derived from the unit weight (reduced mass m = 1000 kg) and the load from the friction of the mower unit slides on the ground and is directed opposite to the movement. This impact is applied uniformly in the form of a concentrated force of 6000 N to the node holding the mower assembly.

### 3.5. Numerical Calculation Results

**Figure 23:**
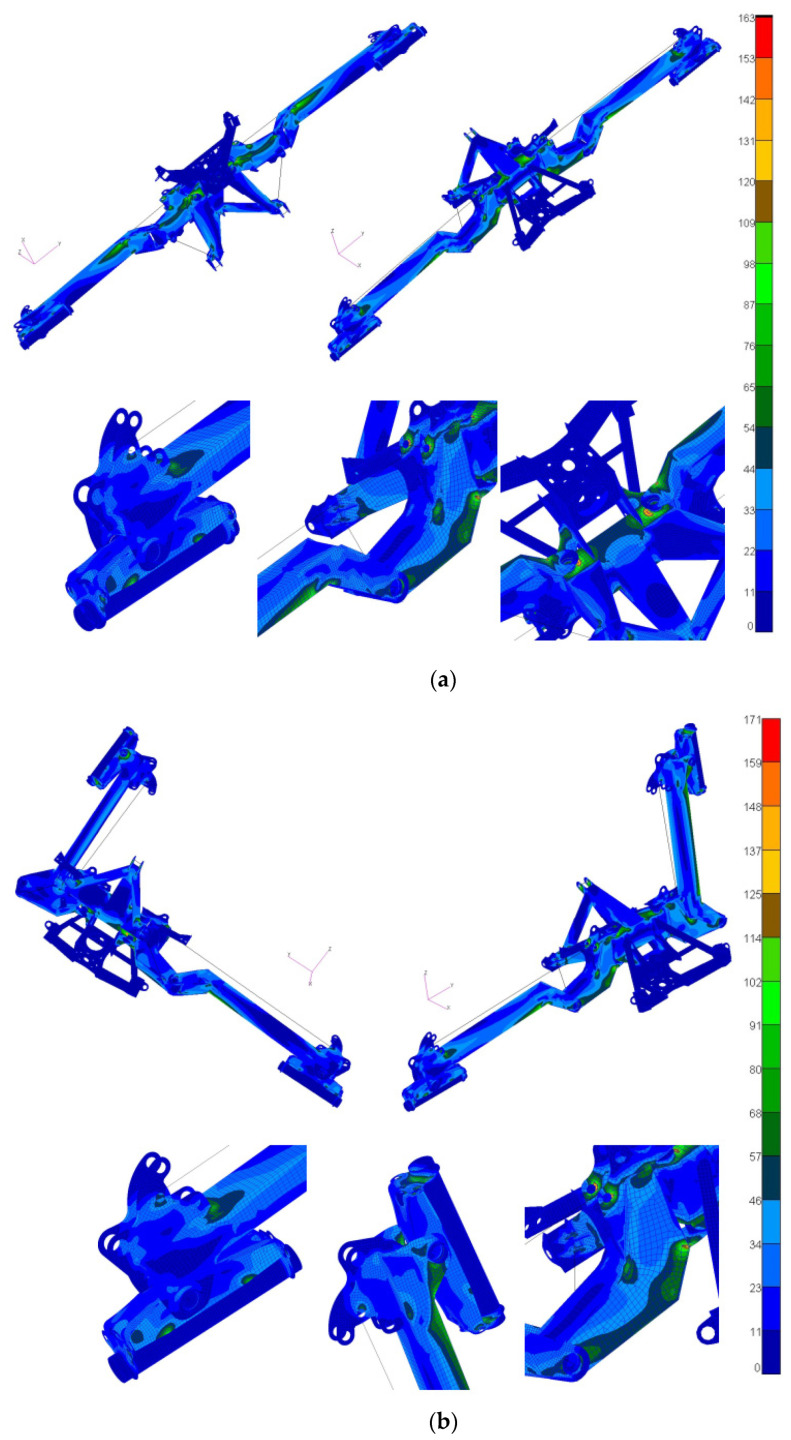
Distribution of equivalent stresses according to HMH: (**a**) configuration 1; (**b**) configuration 2.

As a result of the numerical calculations, the distributions of half stresses of the analyzed structure were determined. The results show the degree of effort of the structure after a given load and illustrate the possibility of using different materials with different strength properties. Figure 23 shows maps of equivalent stresses according to the Huber–Mises–Hencky (HMH) hypothesis obtained for the analyzed configurations. The presented distributions give information about the balanced strain of the mower structure and confirm the validity of using materials with different strength properties in its construction.

## 4. Conclusions

Developing Agriculture 4.0 machinery is vital for boosting agricultural productivity and efficiency, and is especially in alignment with the European Green Deal and related international strategies. High-performance, circular-economy-based machinery should incorporate energy-efficient, carbon footprint-optimized technologies, achieved through thoughtful material selection and low-energy welding. Research into unique welding parameters enhances economic benefits, reduces carbon footprint, and improves operational reliability. Based on the conducted research, the primary conclusions of the study are as follows:

- Utilizing dissimilar welded joints at critical points in green harvesting machine design enhanced structural strength while reducing system weight.
- The non-separable joining of S355 and Strenx 700 steels with the MAG welding method, featuring a 20% reduction in welding parameters based on linear welding energy, significantly increased static joint strength (up to 10%) and flexibility. This improvement was due to reduced thermal energy input, leading to slower grain growth in the weld zone.
- Optimized welding parameters reduced the energy intensity of welding these materials by up to 20%, directly cutting production costs. Using the MAG technique with the adopted welding parameters and the filler metal, both butt and overlay welding of S355 and Strenx 700 steels allowed for the creation of high quality joints concerning macrostructure and strength properties.
- Strength tests revealed that cracking occurred in the heat-affected zone on the S355 steel side.
- The joint exhibited slightly higher strength and elongation values in comparison to the weaker of the joined materials, with a local increase in the joint’s strength limit of up to 10%.
- The use of base material with lower strength characteristics, reinforced by overlays of higher-strength steels, improved the strength and stiffness of the resulting structural element.
- Simultaneously, this approach reduced weight and extended the operational lifespan of the welded joints.
- Implementing such measures in the manufacturing of agricultural machinery reduced production costs and facilitated the design of larger, more efficient, and reliable solutions.
- The recommendations presented in the paper, focusing on the utilization of dissimilar joining materials through optimized welding parameters, resulted in design modifications in Samasz-manufactured agricultural machinery. These modifications specifically targeted the mower, tedder, and rake systems used for multi-row forage harvesting.
- Operational data gathered from these adapted machines revealed a decreased incidence of failures and structural damage, particularly concerning issues related to cracks, in contrast to older solutions.
- This collection of evidence served as concrete validation of the proposals presented in the paper.

## Figures and Tables

**Table 1:** Types of welded joints and the most commonly used thicknesses of joined sheets in the production of a green fodder harvesting machine assembly.

Name	Joint Type	Material 1	Material 2
Sample 1	Butt welds	S355 #5	Strenx 700 #5
Sample 2	Overlay joints	S355 #6	Strenx 700 #5
Sample 3	Overlay joints	S355 #10	Strenx 700 #5
Sample 4	Overlay joints	S355 #12	Strenx 700 #5
Sample 5	Overlay joints	S355 #14	Strenx 700 #5

**Table 2:** The strength properties of the materials to be joined.

Grade of Steel	S355 (A765)	Strenx 700	Hardox 400
Re [MPa]	355	700	1100
Rm [MPa]	520	1100	1250
A5 [%]	22	7	10
Hardness [HB]	220	290	410
	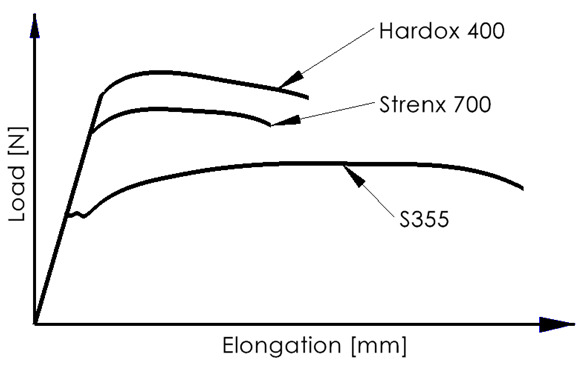		

**Table 3:** Chemical composition of joined materials.

Material	C	Si	Mn	P	S	Al	Cr	Cu	Ni	Mo	B	Nb	V	Ti
Strenx 700	0.11	0.17	2.0	0.018	0.088	0.019	-	-	-	-	-	0.065	0.10	0.11
S355	0.2	0.35	1.5	0.03	0.025	0.016	0.25	0.019	0.2			-	-	-
Hardox 400	0.4	0.4	1.25	0.017	0.01	-	1.34	-	1.35	0.05	0.003	-	-	-

**Table 5:** The strength properties of the joint obtained for different welding parameters.

		Sample 1	Sample 2	Sample 3	Sample 4	Sample 5
Rm [MPa]	V1	564	486	518	542	508
	V2	568	494	521	546	540
	V3	575	496	523	565	553
Re [MPa]	V1	492	410	440	384	440
	V2	496	415	437	388	442
	V3	502	412	445	409	435
A [mm]	V1	3.03	23.5	22.8	24.2	7.63
	V2	2.73	21.40	24.45	23.90	14.82
	V3	3.60	23.70	25.58	27.60	22.22

**Table 6:** Microhardness of dissimilar overlay welded joint for analyzed welding parameters (Sample 4).

No	Measurement Zone	HV20		
		V1	V2	V3
1	Base metal Strenx	268	268	268
2		269	269	270
3	HAZ–Strenx	244	244	240
4		267	267	265
5		271	271	269
6	Weld	240	240	230
7		239	239	228
8		244	244	225
9	HAZ–S355	238	238	230
10		175	175	180
11		168	168	165
12		158	158	157
13		156	156	156
14		156	156	155
15	Base metal S355	152	152	153
16		152	152	151
17		151	151	151
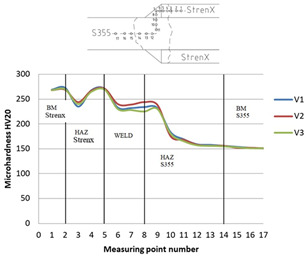				

**Table 7:** Microhardness of dissimilar butt welded joint for analyzed welding parameters (Sample 1).

No	Measurement Zone	HV20		
		V1	V2	V3
1	Base metal Strenx	267	266	267
2		271	270	271
3	HAZ–Strenx	245	242	238
4		271	267	266
5		270	263	265
6	Weld	245	244	236
7		248	245	239
8		243	245	238
9	HAZ–S355	240	241	233
10		183	180	176
11		165	163	158
12	Base metal S355	152	152	153
13		152	152	150
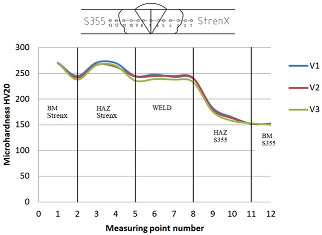				

**Table 8:** Comparison of HAZ size for the analyzed welding parameters of the overlay joint (Sample 4).

Variant	S355	Strenx 700
	HAZ	
V1	100%	100%
V2	95%	90%
V3	75%	80%

## Data Availability

Restrictions apply to the availability of these data. Data were obtained from research work WZ/WM-IIM/4/2020 at the Bialystok University of Technology along with the cooperation of SaMASZ Sp. z o.o. Data are only available by permission of the authors and SaMASZ Sp. z o.o.
